# Enhanced Transepithelial Permeation of Gallic Acid and (−)-Epigallocatechin Gallate across Human Intestinal Caco-2 Cells Using Electrospun Xanthan Nanofibers

**DOI:** 10.3390/pharmaceutics11040155

**Published:** 2019-04-01

**Authors:** Adele Faralli, Elhamalsadat Shekarforoush, Ana C. Mendes, Ioannis S. Chronakis

**Affiliations:** Nano-BioScience Research Group, DTU-Food, Technical University of Denmark, Kemitorvet, B202, 2800 Kgs. Lyngby, Denmark; adele.faralli@gmail.com (A.F.); elham.shekar@gmail.com (E.S.); anac@food.dtu.dk (A.C.M.)

**Keywords:** xanthan gum, electrospinning, gallic acid, (−)-epigallocatechin gallate, permeability

## Abstract

Electrospun xanthan polysaccharide nanofibers (X) were developed as an encapsulation and delivery system of the poorly absorbed polyphenol compounds, gallic acid (GA) and (−)-epigallocatechin gallate (EGCG). Scanning electron microscopy was used to characterize the electrospun nanofibers, and controlled release studies were performed at pH 6.5 and 7.4 in saline buffer, suggesting that the release of polyphenols from xanthan nanofibers follows a non-Fickian mechanism. Furthermore, the X-GA and X-EGCG nanofibers were incubated with Caco-2 cells, and the cell viability, transepithelial transport, and permeability properties across cell monolayers were investigated. Increases of GA and EGCG permeabilities were observed when the polyphenols were loaded into xanthan nanofibers, compared to the free compounds. The observed in vitro permeability enhancement of GA and EGCG was induced by the presence of the polysaccharide nanofibers, which successfully inhibited efflux transporters, as well as by opening tight junctions.

## 1. Introduction

Polyphenols are the most abundant antioxidants in our diet and they are receiving increasing interest due to the established association between the intake of a polyphenol-rich diet and the prevention of various diseases [1,2]. Because of their antioxidant [3], antimutagenic [4], and anticarcinogenic properties [5,6], polyphenols have recently attracted research interest towards the study of their metabolism and absorption mechanisms across the gut barrier [7].

Polyphenols are categorized according to the chemical structure of their carbon skeleton, and the most abundant classes in our diet are phenolic acids and flavonoids. The most encountered phenolic acids are caffeic acid, ferulic acid, and gallic acid (GA). The latter, also known as 3,4,5-trihydroxybenzoic acid, is one of the main endogenous phenolic acids found in plants, mostly in tea leaves [8]. GA, also found in vegetables, grapes, and pomegranates, is a potent non-enzymatic antioxidant and has a natural antitumor activity against lung, prostate, colon, gastric, and breast cancer and human pre-myelocytic leukemia [9,10,11,12]. It has been reported that the in vitro treatment of lung and human cervical cancer cells with GA concentrations in the micromolar range induces cell death associated with the depletion of glutathione (GSH) as well as reactive oxygen species (ROS) level changes [13,14]. The physiological impact and efficiency of GA is strictly dependent on its bioavailability, biochemical integrity, and successful interaction with target tissues. Many studies have demonstrated that only small amounts of orally administered GA are absorbed through the intestine due to its low permeability, poor water solubility, and chemical instability. The GA instability in the gastrointestinal tract is promoted by endogenous enzymes, interfering nutrients, and oxidative reactions that lead to a considerable loss in its activity [15]. It is also reported that the phenol concentrations needed to result in an in vitro efficiency are higher than the moderate in vivo levels, and gastrointestinal permeation is supported only by passive diffusion [15]. Moreover, previous studies found that after oral administration of *Phyllanthi* tannin fraction at a dose of 6 g/kg in rats, the maximum concentration of absorbed GA was less than 10.47 µg/mL [16]. In vitro investigations have also been conducted with Caco-2 cell monolayers, in order to evaluate the transepithelial transport of pure GA across the cellular barrier, and the apparent permeability coefficient, *P_app_*, under a proton gradient was about 0.20 × 10^−6^ cm/s [8]. 

Flavonoids, the most abundant polyphenols in our diet together with phenolic acids, can be divided into several classes, and catechins are the main flavonols found in tea [1]. The major tea catechins are (−)-epigallocatechin gallate (EGCG), (−)-epicatechin gallate (ECG), (−)-epicatechin (EC), and (−)-epigallocatechin (EGC) [17]. These natural compounds have demonstrated various health-beneficial properties, including antioxidant, anti-inflammatory, and anticancer effects, both in animals and humans [18,19]. Indeed, an inverse association between tea consumption and colorectal cancer frequency as well as gastric cancer has been identified [20,21]. An increasing interest towards EGCG has led to an extensive investigation of the beneficial properties of this natural molecule in the cosmetic, nutritional, and pharmaceutical fields. However, like GA, EGCG has a poor oral bioavailability and poor biochemical stability. In fact, EGCG has a low lipophilicity (octanol/water partition coefficient of 0.86 ± 0.03), thus limiting its intrinsic permeability across the intestinal epithelium [19]. Several studies have instead demonstrated a high and specific accumulation of tea flavonoids in epithelial Caco-2 cells or epithelial cells along the aerodigestive tract [17,22,23], which have been recognized as major sites for biological activity of flavonoids. In the Caco-2 cell model, apical uptake transporters and efflux pumps, such as the multidrug resistance-associated proteins, MRP1 and MRP2, and P-glycoprotein have been identified to play a major role in cellular accumulation of catechins [17,19,24,25].

In the light of these considerations, the oral administration of GA and EGCG requires a formulation strategy able to protect and maintain their structural integrity, increase their bioavailability and water solubility, and deliver them to target tissues. Among the existing delivery and stabilization approaches, the encapsulation of sensitive compounds is considered the most effective strategy for improving the oral bioavailability and shelf-life of compounds [15,26,27]. Nowadays, a plethora of encapsulation techniques are commonly used in oral delivery systems, and carrier systems for phenolic acids and flavonoids encapsulation have found feasible approaches to overcome both enzymatic degradation and membrane permeation issues [7,19]. The encapsulation of EGCG in a niosomal formulation results in a significantly enhanced bioactive absorption, stronger chemical stability, and lower toxicity compared with the free EGCG [19]. The in vitro apparent permeability, *P_app_*, of EGCG niosome across Caco-2 cell monolayers was found to be 1.42 ± 0.24 × 10^−6^ cm/s, almost 2-fold more as free EGCG (*P_app_* = 0.88 ± 0.09 × 10^−6^ cm/s). Furthermore, GA-loaded mesoporous silica nanoparticles (MSNs-GA) were easily internalized into Caco-2 cells without any deleterious effect on cell viability, and preserving the same antitumor properties of free GA [7]. In addition, the topical and transdermal delivery of GA loaded into poly(l-lactic acid) fiber mats resulted in a preserved radical scavenging activity of the released phenolic acid [28]. GA has also been encapsulated within electrospun fibers as delivery carriers using the protein zein [29], cellulose acetate [30], and polylactic acid (PLA) nanofibers, including GA-cyclodextrin complexes [31]. The encapsulation and release of EGCG loaded into electrospun nanofibers has also been investigated using zein nanofibers [32], hyaluronic acid/ lactic-*co*-glycolic acid fibers (HA/PLGA, core/shell) [33], PLGA nanofibers [34,35], cellulose electrospun nanofibrous mats coated with bilayers of chitosan and EGCG [36], and electrospun hydroxypropyl methylcellulose nanofibers [37].

In our previous study, electrospun xanthan-chitosan nanofibers loaded with curcumin, as a model hydrophobic bioactive, were incubated with Caco-2 cells and the transepithelial transport and permeability properties across cell monolayers were assessed. A 3.4-fold increase of curcumin permeability was detected in the presence of xanthan-chitosan nanofibers, in comparison with free-curcumin [38,39]. Moreover, electrospun xanthan nanofibers developed from a solution of xanthan dissolved in formic acid, remained intact and morphologically stable over a wide pH range in saline buffers [40]. In the present study, electrospun xanthan nanofibers were assessed as an encapsulation and delivery system of the two polyphenols, GA and EGCG. The xanthan-GA and xanthan-EGCG loaded nanofibers were incubated with Caco-2 cells, and the transepithelial transport and permeability of GA and EGCG across the cell monolayers were investigated.

## 2. Materials and Methods

### 2.1. Materials

The human colon adenocarcinoma cell line, Caco-2 [Caco-2] (ATCC^®^ HTB-37™), was obtained from the American Type Culture Collection (Rockville, MD, USA). Dulbecco’s modified Eagle’s medium (DMEM) high glucose (4.5 g/L), l-glutamine (200 mM), nonessential amino acids (100X), penicillin-streptomycin (10,000 U/mL and 10 mg/mL in 0.9% sodium chloride, respectively), trypsin-EDTA (10X), Dulbecco’s Phosphate Buffered Saline 1X without calcium chloride and magnesium chloride (indicated in the text as PBS), fluorescein sodium salt (FLUO), lucifer yellow dilithium salt (LY), methanesulfonic acid, MES (1 M; pH 5.5–6.7), 4-(2-hydroxyethyl)-1-piperazineethanesulfonic acid solution, HEPES (1 M; pH 7.0–7.6), gallic acid (GA), and (−)-epigallocatechin gallate (EGCG) were purchased from Sigma Aldrich (Brøndby, Denmark). Tissue culture 12-well plates and 12-mm polycarbonate cell culture inserts with an area of 1.12 cm^2^ and a pore size of 0.4 µm were purchased from Corning Costar^®^ Corporation. Fetal bovine serum (FBS) and Hanks’ balanced salt solution (HBSS) with calcium and magnesium and without phenol red were obtained from Thermo Fisher Scientific (Roskilde, Denmark). CellTiter 96^®^ AQueous One Solution Cell Proliferation Assay (MTS) was purchased from Promega Biotech AB (Nacka, Sweden). Xanthan gum (Cosphaderm X-34) from *Xanthomonas campestris* was kindly provided by Cosphatec GmbH (Drehbahn, Hamburg, Germany) [40]. 

### 2.2. Fabrication of Electrospun Nanofibers

Xanthan was dissolved in formic acid at a final concentration of 2.5% *w*/*v* under vigorous stirring overnight at room temperature. Subsequently, GA and EGCG were added to the polysaccharide solution and further stirred for 30 min. The electrospinning setup consisted of a high voltage generator (ES50P-10W, Gamma High Voltage Research, Inc., Ormond Beach, FL, USA) to provide a voltage of 20 kV, and a syringe pump (New Era Pump Systems, Inc., Farmingdale, NY, USA) to feed the xanthan solution at a flow rate of 0.01 mL/min using a 21 G needle gauge. Xanthan fibers were collected on a steel plate covered with an aluminum foil perpendicularly placed at 8 cm from the end of the needle. The electrospinning process was carried out at ambient conditions (20°C and around 20% relative humidity).

### 2.3. Morphology and FTIR Characterisation of the Nanofibers 

The morphology of electrospun X, X-GA, and X-EGCG nanofibers was studied using a Phenom Pro scanning electron microscope (SEM) (Phenom World, Thermo Fisher Scientific, Eindhoven, The Netherlands). For SEM analysis, a small piece of nanofibers web was attached on SEM specimen stubs by a double-sided adhesive tape. The average fiber diameter of nanofibers was calculated using image J analysis software (National Institutes of Health, Bethesda, MD, USA) measured at 100 different points for each image.

Fourier transform infrared (FTIR) spectroscopy analysis of X, X-GA, X-EGCG nanofibers, GA, and EGCG was analyzed using a Perkin Elmer Spectrum 100 spectrometer (Perkin Elmer, Waltham, MA, USA) based on a universal attenuated total reflectance (ATR) sensor. Four scans for each sample were accumulated at 20 °C at a resolution of 1 cm^−1^. The infrared peaks were identified with a Spectrum™ 10 software using a 1% transmittance (T) peak threshold.

### 2.4. In Vitro Release of Gallic Acid and (−)-Epigallocatechin Gallate from Electrospun Nanofibers

The amount of GA and EGCG loaded into xanthan nanofibers was evaluated by immersing the nanofibers in equal volumes of complete growth medium (DMEM-FBS) or HBSS at pH 6.5 or pH 7.4. Briefly, 1.0 mg of X-GA and X-EGCG fibers were immersed in 2 mL pre-warmed medium in a 48-well plate, and the release of molecules from nanofibers was conducted at 37 °C for 8 h. The withdrawn aliquots were analyzed by RP-HPLC with detection of GA and EGCG at 255 nm and 270 nm, respectively (see also Section 2.10). The cumulative amount of each compound released from nanofibers was then considered as the maximum releasable GA and EGCG from the nanofiber formulation under those conditions. All data are expressed as mean ± SD of three independent experiments.

### 2.5. Caco-2 Cell Culture and Subculture

Caco-2 cells were routinely seeded at a concentration of 1.0 × 10^5^ cells/mL in T-75 cm^2^ flasks and incubated at 37 °C in a humidified atmosphere of 5% CO_2_. The complete cell medium, here indicated as DMEM-FBS, consisted of high glucose DMEM containing 10% heat-inactivated FBS, 2 mM l-glutamine, 1% nonessential amino acids, 100 U/mL penicillin, and 100 µg/mL streptomycin. The medium was renewed every second day until cells reached approximately 90% confluence. Cells were passaged at a subcultivation ratio of 1:4 by treatment with 0.25% trypsin—0.53 mM EDTA solution for 10 min at 37 °C. After trypsinization, the cells were suspended in complete growth medium and centrifuged for 5 min at 1000 rpm. After supernatant removal, the pellet was suspended in the growth medium and cell concentration was determined with an ORFLO Moxi Z Mini Automated Cell Counter using Type S cassette (Biofrontier Technology, Bukit, Singapore). All Caco-2 cells were used between passages 9–15.

### 2.6. Compounds and Electrospun Nanofibers Tested with Caco-2 Cell Monolayers

Xanthan (X), gallic acid-loaded xanthan (X-GA), and (−)-epigallocatechin gallate-loaded xanthan (X-EGCG) nanofibers were produced by electrospinning a solution of the mixed compounds dissolved in formic acid. These nanofibers were tested with Caco-2 cell monolayers to evaluate their toxicity and apparent permeability coefficient (P_app_) after GA and EGCG release from nanofibers and as free compounds. Before testing nanofiber mats with Caco-2 cells, the collected fibers were kept under an air stream for 3 days allowing complete formic acid evaporation. Besides GA and EGCG, the transepithelial transport of fluorescein (FLUO) and Lucifer yellow (LY) across Caco-2 cell monolayers were also investigated as markers for intestinal epithelial permeability and integrity.

### 2.7. Caco-2 Cell Viability Assay 

The in vitro Caco-2 cell viability after treatment with free GA, free EGCG, xanthan nanofibers (X), GA-loaded xanthan nanofibers (X-GA), and EGCG-loaded xanthan nanofibers was evaluated by using the MTS [3-(4,5-dimethylthiazol-2-yl)-5-(3-carboxymethoxyphenyl)-2-(4-sulfophenyl)-2H-tetrazolium inner salt] colorimetric bioassay. Different concentrations of free GA and EGCG ranging from 1 µM to 1 mM were prepared in PBS and sterile-filtered with a 0.22 µm pore size. Furthermore, increasing amounts of dried X, X-GA, and X-EGCG nanofibers were peeled off from the aluminum foils and incubated with cells. In a 48-well plate, 1.5 × 10^5^ cells/mL were seeded in a complete growth medium and incubated for 2 days at 37 °C in a humidified atmosphere of 5% CO_2_. Then, the monolayers were washed with PBS and the complete medium was renewed. Caco-2 cells were incubated with free GA and free EGCG solutions, X nanofibers, X-GA nanofibers, X-EGCG nanofibers, and PBS as a control. The plates were incubated for 24 h at 37 °C in a humidified atmosphere of 5% CO_2_. The following day, all supernatants, including those with suspended nanofibers, were removed, cells were washed with PBS, and the medium was renewed. 40 µL of pre-warmed MTS solution was added to each well under dark conditions. After 3 h incubation at 37 °C, the absorbance of the reduced MTS (formazan product) was recorded at 490 nm through a well plate reader (Wallac 1420 Victor2 Multilabel Counter, Perkin Elmer, Waltham, MA, USA).

### 2.8. Transepithelial Transport 

The transepithelial transport of free fluorescein (FLUO), free lucifer yellow (LY), free gallic acid (GA), free (−)-epigallocatechin gallate (EGCG), free gallic acid in the presence of empty xanthan nanofibers (X + GA), free (−)-epigallocatechin gallate in the presence of empty xanthan nanofibers (X + EGCG), gallic acid-loaded xanthan nanofibers (X-GA), and (−)-epigallocatechin gallate-loaded xanthan nanofibers (X-EGCG) across Caco-2 cell monolayers were investigated according to the protocol reported by Hubatsch et al. [41]. The transport experiments were performed in both apical-to-basolateral (AB, absorptive) and basolateral-to-apical (BA, secretory) directions, under a proton gradient. In fact, to mimic the acidic microclimate of the small intestine, apical and basolateral pH of around 6.5 and 7.4 were used, respectively. Briefly, 1.0 × 10^5^ cells/insert were seeded onto pre-wetted 12-mm polycarbonate cell culture inserts with an area of 1.12 cm^2^ and a pore size of 0.4 µm. The apical and basolateral compartments were filled with 0.5 mL and 1.5 mL complete medium, respectively. The Caco-2 cells were incubated onto the filters overnight at 37 °C in a humidified atmosphere of 5% CO_2_. The day after, the growth medium was replaced in both compartments and the plates were incubated for 21 days at 37 °C in a humidified atmosphere of 5% CO_2_, renewing the complete growth medium every second day. For the AB transport experiments, donor solutions of FLUO, LY, GA, and EGCG at a concentration of 11 mM, 9.57 mM, 1.1 mM, and 1.1 mM, respectively, were prepared in sterile-filter HBSS at pH 6.5 buffered with 10 mM MES. Again, donor solutions of FLUO, LY, GA, and EGCG at a concentration of 10.3 mM, 9 mM, 1.03 mM, and 1.03 mM, respectively, were prepared in sterile-filter HBSS at pH 7.4 buffered with 25 mM HEPES to evaluate their BA transport. A volume of 50 µL of each stock solution was added to the donor chamber (0.55 mL and 1.55 mL were the total volumes in A and B, respectively). The transport of GA and EGCG released from nanofibers and as free compounds in the presence of empty X nanofibers was also investigated. For the AB transport, 0.2 mg X-GA, 1.0 mg X-EGCG, 0.2 mg, and 1.0 mg X were used, and accordingly, 0.6 mg X-GA, 3.0 mg X-EGCG, 0.6 mg, and 3.0 mg X were incubated with cell monolayers to evaluate their BA transport. Prior to incubation of the nanofibers, the mats were peeled off from the aluminum foil and kept under an air stream for 3 days. After 21 days of cell growth, the complete DMEM medium was removed from the cell monolayers and replaced with HBSS at pH 6.5 and pH 7.4 at the apical and basolateral compartments, respectively. For the AB transport studies, 1.5 mL HBSS was used in the basolateral side and 0.55 mL of each donor solution and/or nanofibers were added to the apical side. Immediately, 200 µL aliquots were withdrawn from each donor compartment (time = 0). Aliquots from the acceptor side were then withdrawn at different time intervals, and the volume was replaced with fresh HBSS at pH 7.4 maintaining the well plates at 37 °C in a humidified atmosphere of 5% CO_2_. A final aliquot from the donor chamber was taken as the last time point. BA transport studies were conducted using the same procedure and incubating 0.5 mL HBSS at pH 6.5 in the apical side and 1.55 mL of donor solution and/or nanofibers in the basolateral chamber. During the transport experiments, all cell media were pre-warmed at 37 °C. Each transport experiment was performed for a time interval of 8 h in triplicate (*n* = 3). After 8 h of transport studies and TEER measurements, both apical and basolateral chambers were washed twice with PBS and cell monolayers were detached from the insert membrane with 0.25% trypsin-0.53 mM EDTA solution for 10 min at 37 °C. The collected Caco-2 cell lysates were centrifuged for 5 min at 1000 rpm and supernatants were discarded. Furthermore, the semipermeable membranes were carefully removed from the insert using a scalpel and collected into Eppendorf tubes in 500 µL HBSS at pH 6.5 (apical conditions). Cell pellets were re-suspended in 500 µL HBSS at pH 6.5 and both cells and membranes were sonicated for 3 h using an ultrasonic bath (Branson Ultrasonic Corp., VWR, Søborg, Denmark). The collected samples were then centrifuged for 15 min at 10,000 rpm and supernatants were analyzed by HPLC (Thermo Fisher Scientific, Roskilde, Denmark). The same procedure was used to quantify the compound amounts adsorbed (X + GA and X + EGCG) or remaining encapsulated (X-GA and X-EGCG) in the nanofibers at the end of the transport experiments. The tested nanofibers were removed from the donor chamber and suspended in 500 µL of HBSS (pH 6.5 for AB transport and pH 7.4 for BA transport). After sonication and centrifugation, the molecules in the supernatants were quantified by HPLC.

### 2.9. Measurement of Transepithelial Electrical Resistance (TEER) 

The transepithelial electrical resistance (TEER) was measured at 20 °C before and after permeability experiments with an epithelial volt-ohmmeter equipped with STX2 “chopstick” electrodes (EVOM2™, World Precision Instruments, Sarasota, FL, USA). Before measuring the resistance values of each well, the cell monolayers and the basolateral chamber were washed twice with pre-warmed HBSS at pH 6.5 and HBSS at pH 7.4, respectively. The resistance values of the semipermeable membrane without cells (R_BLANK_) were recorded and subtracted from the resistance values obtained from the measurement of each cellular monolayer onto the semipermeable membrane (R_TOTAL_). The specific cell resistance values (R_TISSUE_) were calculated by:

R_TISSUE_ (Ω) = R_TOTAL_ (Ω) − R_BLANK_ (Ω) (1)

TEER values of cellular monolayers were expressed in Ω × cm^2^ and calculated by:

TEER_TISSUE_ (Ω cm^2^) = R_TISSUE_ (Ω) × A_MEMBRANE_ (cm^2^) (2)

### 2.10. Quantification of Compounds

Donor solutions of FLUO, LY, GA, and EGCG were prepared and sterile-filtered in HBSS at pH 6.5 and pH 7.4 to perform transepithelial studies. Standard curves of GA and EGCG dissolved in HBSS at pH 6.5 and pH 7.4 were obtained by HPLC analysis. 200 µL samples withdrawn from the donor and acceptor compartments during transport experiments across cell monolayers were quantitatively analyzed using RP-HPLC (Thermo Fisher Scientific, Denmark). A C18 column (3.0 × 100 mm) and 0.5 mL/min flow rate were used. GA and EGCG were quantified with detection at 255 nm and 270 nm, respectively. FLUO and LY aliquots were instead analyzed by UV-vis spectrometry (Nanodrop One^C^, Thermo Fisher Scientific, Denmark), recording their absorbance at 490 nm and 430 nm, respectively. The amount of each compound transported across the cell monolayers within a time interval of 8 h was calculated for both apical-to-basolateral (AB) and basolateral-to-apical (BA) directions. FLUO, LY, GA, and EGCG that remained entrapped within the cell monolayers, insert membranes and nanofibers were likewise quantified at the end of the permeability studies.

### 2.11. FLUO, LY, GA, and EGCG Distribution after Transport Experiments and Mass Balance

After transport experiments in both AB and BA directions, the amount of each compound collected at the apical and basolateral chambers was quantified. Donor concentrations at time = 0 (C_D,t=0 h_), donor and acceptor concentrations at time = 8 h (C_D,t=8 h_ and C_A,t=8 h_), compound concentrations remaining inside the cell monolayer at time = 8 h (C_Caco-2,t=8 h_), within membrane filters at time = 8 h (C_insert,t=8 h_), and adsorbed or remaining encapsulated in nanofibers at time = 8 h (C_fibers,t=8 h_) were experimentally measured. The mass balance of each compound was calculated as follows:

(3) $$C_{D,t=0\;h}=C_{D,t=8\;h}+{\;C}_{A,t\;=\;8\;h}+{\;C}_{\mathrm{Caco}-2,t=8\;h}+{\;C}_{\mathrm{insert},t=8\;h}+\;\left(C_{\mathrm{fibers},t=8\;h}\right)$$

Mass balance values of >90% were found for all tested compounds.

### 2.12. Calculation of the Apparent Permeability Coefficients, P_app,AB_ and P_app,BA_

The absorptive apparent permeability coefficient (P_app,AB_) and the secretory apparent permeability coefficient (P_app,BA_) were calculated by:

(4) $$P_{\mathrm{app}}=\;\frac{\mathrm{dC}}{\mathrm{dt}}*\;\frac{V}{{A*C}_{0}}$$

where, *dC/dt* (µM/s) is the rate of change in concentration on the acceptor chamber over time; *V* (cm^3^) is the volume of the solution in the acceptor compartment; *A* (cm^2^) is the area of the semipermeable membrane; and *C*_0_ (µM) is the initial concentration in the donor chamber. The results of this study are expressed as mean ± SD of three independent experiments. Permeability directional ratio (PDR) is a measure of the compound polarization in Caco-2 cell monolayers, and was calculated by:

(5) $$\mathrm{PDR}=\;\frac{P_{\mathrm{app},\mathrm{BA}}}{P_{\mathrm{app},\mathrm{AB}}}$$

## 3. Results 

### 3.1. Morphological and FTIR Characterization of Nanofibers 

**Figure 1:**
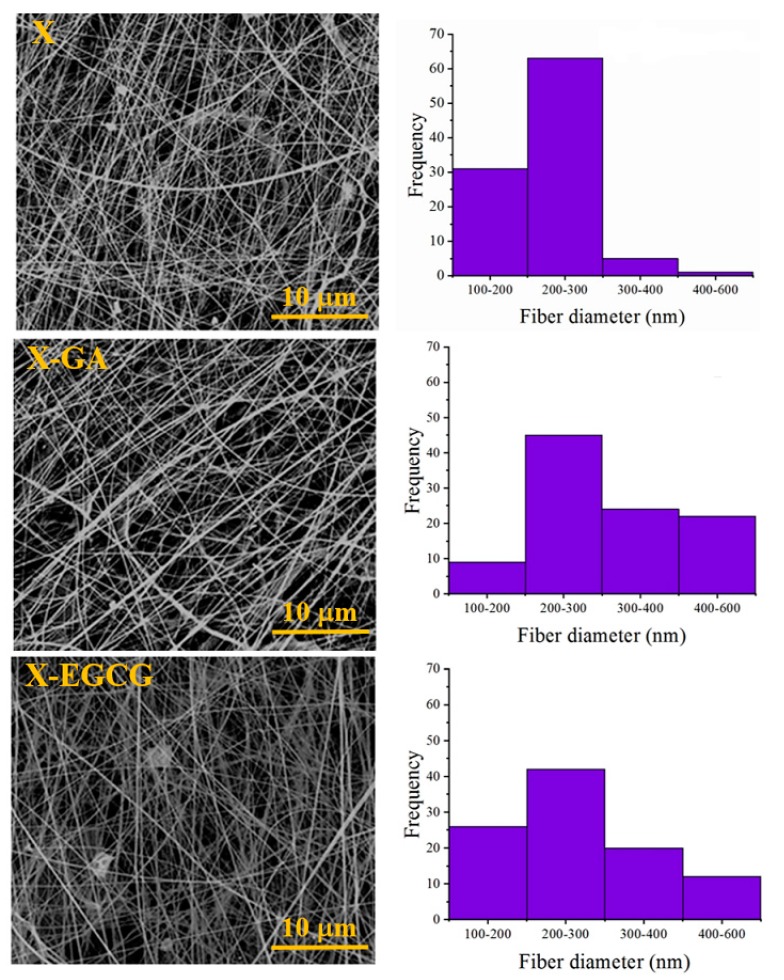
Morphological analysis by scanning electron microscopy (SEM) and average fiber diameter distributions of electrospun (upper) X nanofibers, (middle) X-GA, and (lower) X-EGCG nanofibers.

Uniform and randomly oriented xanthan nanofibers, with average diameters of 235 ± 49 nm, were obtained by electrospinning a 2.5% *w*/*v* xanthan solution in formic acid (Figure 1). The average diameter of electrospun X-GA and X-EGCG nanofibers was slightly increased to 327 ± 119 nm and 270 ± 95 nm, respectively, with the encapsulation of 2 mM of phenolic compounds.

**Figure 2:**
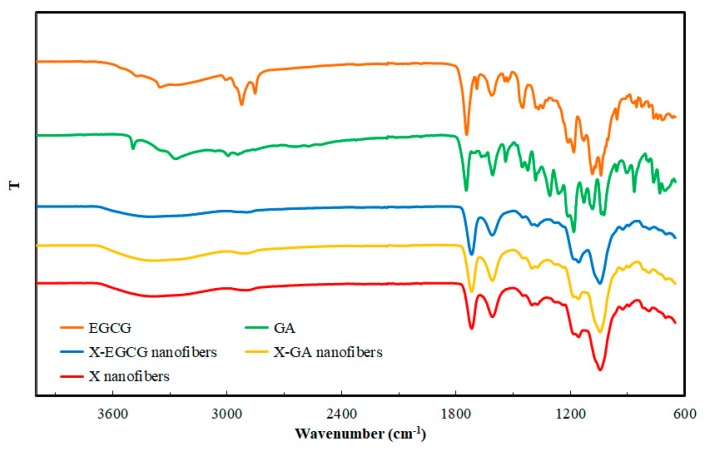
FTIR spectra of electrospun X, X-GA, X-EGCG nanofibers, GA, and EGCG.

The FTIR spectra of X nanofibers, X-GA nanofibers, X-EGCG nanofibers, and GA and EGCG powders are shown in Figure 2. The FTIR spectrum of X nanofibers showed a characteristic broad peak in the region of 3500–3000 cm^−1^ due to O-H stretching, and a peak at around 2900 cm^−1^ due to the axial deformation of CH and CH_2_ groups. In the region between 1800–1700 cm^−1^, the stretching vibration of C=O was observed. In the region of 1200–1000 cm^−1^, the O–H, C–O–C stretching of tertiary alcohols and esters, as well as the O–H stretching of primary alcohols was distinguished [40]. As discussed in the study by Shekarforoush et al. [40], the FTIR studies confirmed that an esterification reaction had taken place, where formic acid reacted with the pyruvic acid groups of xanthan. Hence, the esterification of pyruvic acid to pyruvyl formate induced a decrease of the negative charges of xanthan and stabilized the helical conformation of xanthan. Moreover, the FTIR spectra of X-GA and X-EGCG nanofibers are comparable to those of X without the bioactives. It is concluded that there are no physical or chemical interactions between the encapsulated GA, EGCG, and the X nanofibers matrix.

### 3.2. In Vitro Release of GA and EGCG from Xanthan Nanofibers

**Figure 3:**
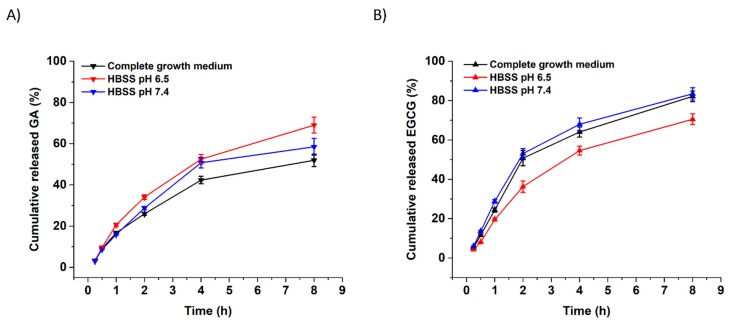
Cumulative in vitro release of GA (**A**) and EGCG (**B**) from xanthan nanofibers. All data were the mean ± SD of three independent experiments.

The cumulative in vitro release of GA and EGCG from xanthan nanofibers was investigated by immersing the fibers in complete growth medium (DMEM-FBS), HBSS at pH 6.5 and HBSS at pH 7.4 (Figure 3). The total amount of GA and EGCG released from fibers was 69.01% and 70.53% in HBSS at pH 6.5, and 58.47% and 83.44% in HBSS at pH 7.4, respectively. Slightly different release values emerged from the immersion of fibers in the complete growth medium, which had an experimentally measured pH value of 7.28. Electrospun X, X-GA, and X-EGCG nanofibers remained intact in all release media and no morphological changes were observed during the experimental studies (data not shown). It is suggested that the presence of several salts in both DMEM-FBS and HBSS successfully prevented the dissolution of X, X-GA, and X-EGCG nanofibers.

The mechanism of GA and EGCG release from X nanofibers in pH 6.5 and 7.4 media were fitted by a Korsmeyer-Peppas kinetic model ($C=kt^{n}$), where, *C* is the amount of the compound released within the time, *t*; *k* is the rate constant; and *n* the release exponent. The constant value of *k* is usually related to the characteristics of the delivery system and drug; while *n* is the diffusion exponent, which characterizes the transport mechanism of the compound, and it depends on the type of transport, geometry, and polydispersity. The *n* values of the kinetic model in pH 6.5 and 7.4 media for the release of GA were 0.85 and 0.83, respectively. In the case of EGCG release, the *n* values in pH 6.5 and 7.4 media were 0.84 and 0.77, respectively. These results confirm that the release of the studied phenolic compounds is governed by the non-Fickian mechanism.

### 3.3. Effect of GA, EGCG, and Their Nanofiber Forms on Caco-2 Cell Viability 

**Figure 4:**
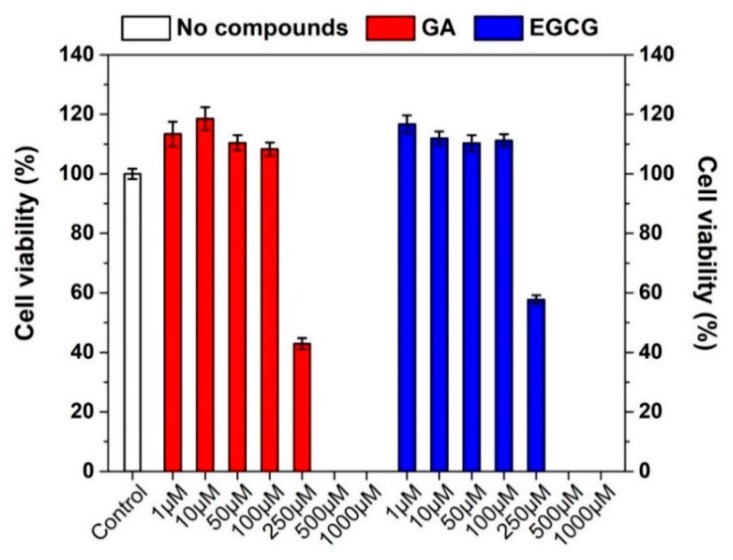
Viability bioassay of Caco-2 cells incubated with PBS (control, white bar) and increasing concentrations of free GA (red bars) and free EGCG (blue bars) diluted in PBS ranging from 1 µM to 1 mM. Data were the mean ± SD of four independent experiments.

The viability of Caco-2 cells after 24 h treatment with free GA, EGCG, and PBS as the control was evaluated through MTS bioassay (Figure 4). 

When Caco-2 cells were incubated with free GA or EGCG in the concentration range between 1–100 µM, an increase in the cell viability was observed. By contrast, concentrations above 100 µM resulted in a drastically decreased cell viability, with a 50% or even higher cell mortality. The IC_50_ of free GA after 24 h incubation was estimated to be around 180 µM [7]. The concentration-dependent toxic effect of GA and EGCG was fundamental to perform transepithelial transport studies across proliferating cell monolayers. Indeed, the amount of X-GA and X-EGCG fibers was accordingly selected to obtain a final released GA and EGCG concentration lower or equal to 100 µM. 

**Figure 5:**
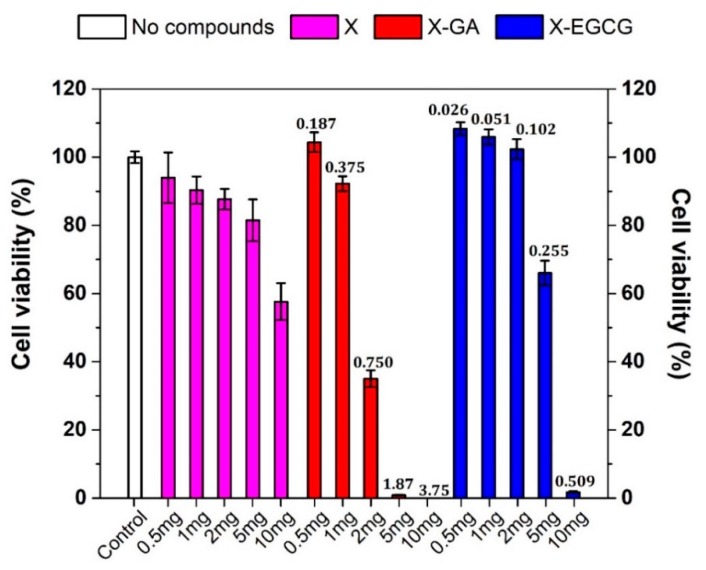
MTS viability bioassay of Caco-2 cells after 24 h incubation with complete growth medium (control, white bar) and increasing amounts of empty xanthan nanofibers (X, magenta bars), gallic acid-loaded xanthan nanofibers (X-GA, red bars), and (−)-epigallocatechin gallate-loaded xanthan nanofibers (X-EGCG, blue bars). The numbers reported on top of the red and blue bars represent the maximum releasable concentration (mM) of GA and EGCG in a 1.2 mL volume of complete growth medium. Data were the mean ± SD of four independent experiments.

The viability of Caco-2 cells after 24 h treatment with increasing amounts of empty X, X-GA, and X-EGCG nanofibers was also investigated to establish the amount of fibers (in milligrams) to be used for transepithelial transport studies. As shown in Figure 5, the incubation of empty X fibers induced a directly proportional decrease of cell viability, reaching around 60% cell viability for 10 mg X nanofibers. However, this reduction was found to be more pronounced when cells were treated with X-GA and X-EGCG fibers. The release of GA from 2.0 mg X-GA fibers caused a cell mortality of around 70% and down until 98% for 5 mg X-GA fibers. The same effect was also confirmed after EGCG release from X-EGCG fibers, even though a 95% cell mortality was observed for 10 mg fibers. Consequently, the reduction of cell viability induced by X-GA and X-EGCG fibers was mainly attributed to GA and EGCG release, as confirmed in Figure 4, and partially caused by X nanofibers. Transepithelial transport studies were conducted incubating in 0.4 mg/mL X-GA (corresponding to 0.15 mM GA) and 1 mg/mL X-EGCG (corresponding to 0.051mM EGCG) in the donor chamber.

### 3.4. Assessment of Cell Monolayers’ Integrity

**Figure 6:**
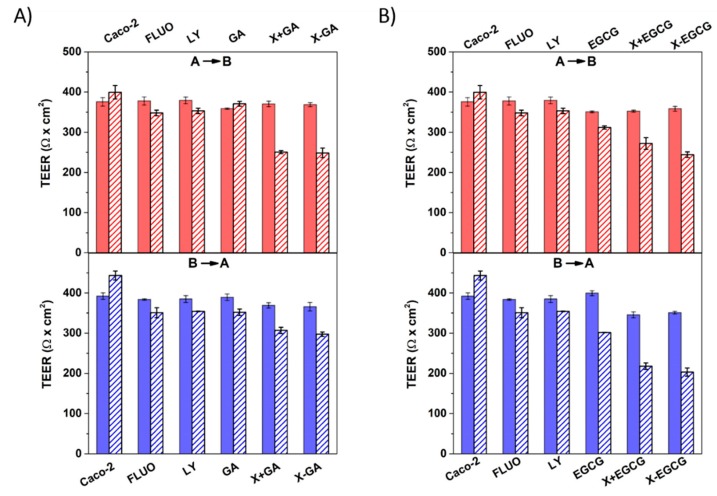
Transepithelial electrical resistance (TEER) measurements of cell monolayers before (full colored bars) and after (patterned bars) apical-to-basolateral (AB) and basolateral-to-apical (BA) studies for a time interval of 8 h. TEER values were recorded for GA, X + GA, and X-GA (**A**) and EGCG, X + EGCG, and X-EGCG (**B**). All data were the mean ± SD of three independent experiments.

The cell monolayers’ integrity is a fundamental determinant for the study of compound transport across the intestinal barrier, especially when passive transport through tight junctions is involved [42]. To ensure reliable in vitro permeability experiments across Caco-2 cell monolayers, the transport of non-radiolabeled markers, fluorescein (FLUO) and lucifer yellow (LY), and transepithelial electrical resistance measurement were conducted to quantitatively investigate the integrity of monolayers after 21 days growth on 12-mm polycarbonate inserts. The average TEER value for Caco-2 cell monolayers randomly chosen for transport studies was 370.74 ± 15.81 Ω cm^2^. The TEER values of monolayers before and after transport of FLUO and LY were found in the range of 300–500 Ω cm^2^ (Figure 6), indicating an “intermediate” tightness of the gastrointestinal epithelium [43].

**Figure 7:**
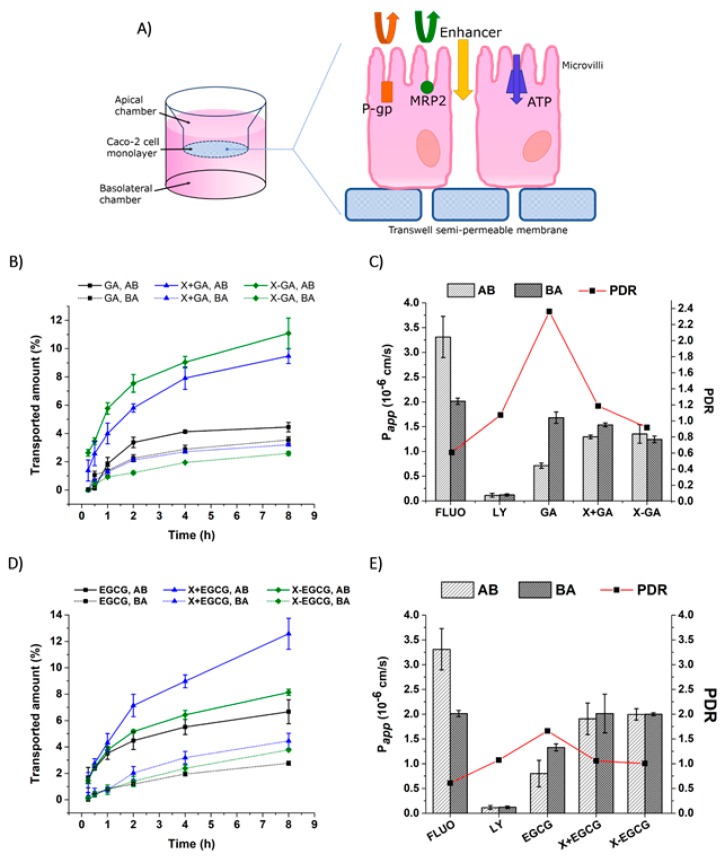
Transepithelial transport of GA and EGCG across Caco-2 monolayers. Illustration of the efflux transporters expressed on the apical membrane of epithelial cells (**A**). Transported amount of GA, X + GA, and X-GA (**B**), and EGCG, X + EGCG, and X-EGCG (**D**) in both AB and BA directions. Apparent permeability coefficient, *P_app_*, and PDR of GA, X + GA and X-GA (**C**), and EGCG, X + EGCG, and X-EGCG (**E**). All data were the mean ± SD of three independent experiments.

The AB and BA transepithelial transports of FLUO and LY across Caco-2 monolayers under a proton gradient were investigated, resulting in a pH-dependent transport of FLUO. The apparent permeability coefficients of FLUO were P_app,AB_ = 3.31 × 10^−6^ cm/s and P_app,BA_ = 2.01 × 10^−6^ cm/s, whereas much lower values were observed from the LY transport: P_app,AB_ = 1.13 × 10^−7^ cm/s and P_app,BA_ = 1.21 × 10^−7^ cm/s (Figure 7C). Due to the lipoid nature of polarized epithelial cell layers, the transport of ions and hydrophilic compounds is restricted through the membrane. Indeed, hydrophilic LY was transported across epithelial cells solely via tight junctions, whereas the lipophilic FLUO permeated through transcellular transport [44,45,46]. Thus, from the TEER and permeability observations it was concluded that the integrity and tightness of epithelial cell monolayers were maintained after 21 days culturing.

### 3.5. Transepithelial Transport and Distribution of Free GA, EGCG, and Their Nanofiber Forms

The transported amounts of GA and EGCG, their apparent permeability coefficient, and their permeability directional ratio were assessed for both the AB and BA directions under a proton gradient. In addition, the compounds were incubated at the donor chamber in a free form (GA and EGCG), in a free form in the presence of empty xanthan nanofibers (X + GA and X + EGCG), and in the nanofiber forms (X-GA and X-EGCG). Figure 7 summarizes all the above-mentioned parameters. Firstly, the amounts of molecules transported in the acceptor chamber were higher in the AB direction than in the BA. Secondly, the addition of empty or loaded xanthan nanofibers enhanced the transport of GA and EGCG in the AB direction (Figure 7B–D). Indeed, the permeated amount of GA in the X + GA and X-GA formulations was 2-fold and 2.5-fold higher than that of free GA. The same results were obtained for the transported EGCG in the AB direction, but on the contrary, the X + EGCG form was the most effective (1.9-fold increase over the free EGCG). These results suggested that the permeation of the compounds was greatly enhanced by the presence of xanthan nanofibers, either as empty nanostructures or loaded with polyphenols. Accordingly, the apparent permeability coefficients of GA and EGCG incubated with nanofibers were at least 2-fold higher than those of the free compounds. Indeed, the GA and X-GA permeability values in the AB direction were P_app,AB_ = 7.12 × 10^−7^ cm/s and P_app,AB_ = 1.96 × 10^−6^ cm/s, respectively (Figure 7C). The same increase in permeability was detected also for the EGCG nanofiber form, where EGCG and X-EGCG had a P_app,AB_ = 7.99 × 10^−7^ cm/s and P_app,AB_ = 1.99 × 10^−6^ cm/s, respectively (Figure 7E). An increment of the apparent permeability coefficient values was also found in the BA direction, even though this was less pronounced than in the AB direction. 

**Figure 8:**
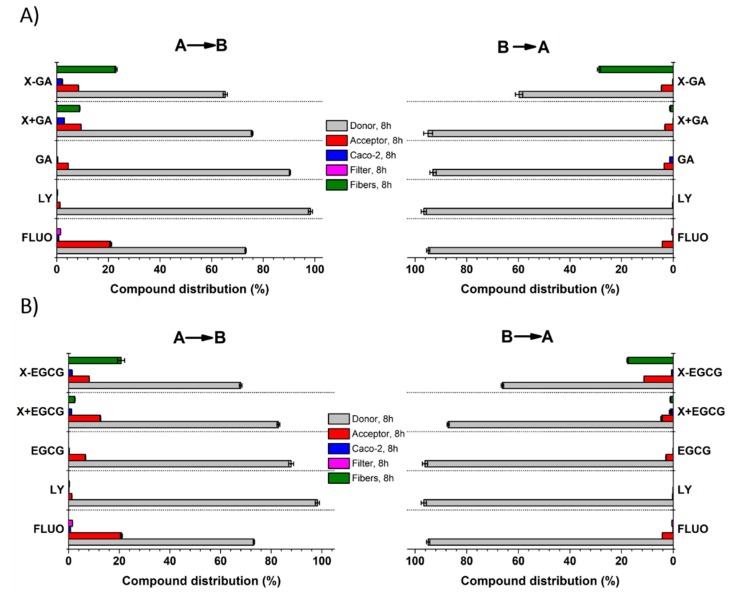
Quantification of GA (**A**) and EGCG (**B**) distribution in the donor side, acceptor side, cell lysate, membrane insert, and fibers after 8 h transepithelial transport in both AB and BA directions. All data are the mean ± SD of three independent experiments.

The fate of GA and EGCG during 8 h transepithelial transport in both AB and BA directions, was monitored by quantifying their concentration in the donor and acceptor compartments, in the cell lysate, insert membrane (filter), and within xanthan nanofibers (adsorbed or unreleased). Figure 8 shows the distribution of the tested compounds in the above-mentioned compartments. After 8 h experiment, most of the incubated compounds were still found in the donor chamber (≥60% of the concentration at time = 0 h), and only less than 20% were detected in the acceptor side. However, the yields of GA and EGCG recorded in A were higher when incubated with xanthan nanofibers than in its absence. Small amounts of GA and EGCG were also detected inside the epithelial monolayers (3% and 1.3%, respectively), and adsorbed to or unreleased from xanthan nanofibers (29% and 21%, respectively). 

## 4. Discussion

In this study, human differentiated epithelial Caco-2 cells were chosen as an established in vitro cell model for the prediction of bioactive compounds’ absorption and transport mechanism [47]. The Caco-2 cells possess many features, among which are their ability to slowly differentiate into monolayers forming microvilli and tight junctions at the apical side, and to express brush-border transporters and enzymes involved in the metabolism and transport of several substrates [41,48,49]. Therefore, transepithelial transport studies of GA and EGCG were performed across Caco-2 monolayers in the apical-to-basolateral and basolateral-to-apical directions under a proton gradient. The two polyphenols investigated in this study are characterized by a poor intestinal absorption due to their high hydrophilicity; in fact, they can hardly penetrate the cell membrane and only passive diffusion seems to be involved in their permeation [19]. 

The incubation of nanofibers with Caco-2 cells (24 h) revealed a proliferative effect in cell viability for amounts lower or equal to 0.5 mg X-GA and 2.0 mg X-EGCG; a drastic cell mortality was observed for doses above this range. In addition, the treatment of Caco-2 cells with increasing amounts of empty xanthan nanofibers resulted in a dose-dependent reduction of cell viability, close to 60% viability for 10.0 mg X incubated. However, the observed reduction in cell viability was expected to be less pronounced for transepithelial transport studies, since the cell monolayers were exposed to X, X-GA, and X-EGCG for 8 h rather than 24 h. The transepithelial transport of GA and EGCG in the acceptor compartment was successfully enhanced by the presence of xanthan, both as an empty nanostructure and as a nanocarrier, and the permeability coefficients were higher than those calculated for free compounds. In addition, the PDR values of free GA and free EGCG were both higher than 1.5 (2.4 and 1.7, respectively), suggesting that their transport is modulated by an active transport pathway, and more specifically by efflux. Several studies have described the mechanism and the efflux transporters involved in the unidirectional transport of GA and EGCG across the epithelial barrier [16,17,19,50,51]. Enterocytes express several transporters on the apical and basolateral membrane, which can actively transport a wide range of structurally diverse compounds into (influx) or out of (efflux) the cell. GA and EGCG, as depicted in Figure 7A, are actively transported outside cells through P-glycoprotein (P-gp), multidrug resistant protein 2 (MRP2), and the ATP binding cassette (ATP) transporters expressed on the apical membrane of Caco-2 monolayers [49,51]. These efflux pumps, therefore, restrict the influx of GA and EGCG into the acceptor chamber, rather promoting their efflux from enterocytes. Several efflux pump inhibitory compounds, such as indomethacin, verapamil, and MK-571 [16,19], have been thoroughly investigated, resulting in an increase in oral absorption. In this study, the calculated PDR values obtained for free GA and free EGCG transport were higher than 1.5, confirming their efflux from monolayers. However, the PDR values of X + GA, X-GA, X + EGCG, and X-EGCG were all lower than 1.5 (Figure 7C–E). Hence, the incubation of xanthan nanofibers in the donor compartment greatly improved the absorption of GA and EGCG across the epithelial barrier, suggesting an inhibitory effect of xanthan on efflux transporters. 

The results presented in this study are congruent with our previous findings on the permeation across Caco-2 cells of a model protein (insulin) encapsulated within electrospun fish protein fibers [52]. Direct interactions between the fibers and the monolayer induced changes in the tight junctions, and thus, an increase in the permeation of insulin at local hot spots on the epithelial barrier was observed. Similarly, a 3.4-fold increase of curcumin permeability across Caco-2 cells was detected when the bioactive was encapsulated within xanthan-chitosan nanofibers, in comparison with free-curcumin [39].

## 5. Conclusions

Encapsulation and release of two poorly absorbed polyphenol compounds, GA and EGCG, using electrospun xanthan nanofibers were investigated. It was found that X, X-GA, and X-EGCG nanofibers remained stable in aqueous HBSS medium at different pH (6.5 and pH 7.4). The total amount of GA and EGCG released from xanthan nanofibers was 69.01% and 70.53% in HBSS at pH 6.5, and 58.47% and 83.44% in HBSS at pH 7.4, respectively. Moreover, the nanofibers were incubated with Caco-2 cells and the cell viability, transepithelial transport, and GA and EGCG permeability properties across cell monolayers were investigated. At least a 2-fold increase of GA and EGCG permeability was observed in the presence of X-GA and X-EGCG nanofibers, in comparison with the free-phenolic compounds. Indeed, the polysaccharide nanofibers enhanced the GA and EGCG permeability by opening the tight junctions of Caco-2 monolayers, as well as inhibiting the efflux transporters. These findings are relevant for promoting the delivery not only of polyphenols, but also of other poorly absorbable bioactives and drugs.
